# Impact of stress on oocyte quality and reproductive outcome

**DOI:** 10.1186/s12929-016-0253-4

**Published:** 2016-03-29

**Authors:** Shilpa Prasad, Meenakshi Tiwari, Ashutosh N. Pandey, Tulsidas G. Shrivastav, Shail K. Chaube

**Affiliations:** Cell Physiology Laboratory, Biochemistry Unit, Department of Zoology, Institute of Science, Banaras Hindu University, Varanasi, 221005 UP India; Department of Reproductive Biomedicine, National Institute of Health and Family Welfare, Baba Gang Nath Marg, Munirka, New Delhi, 110067 India

**Keywords:** Stress, ROS, Oxidative stress, Oocyte quality, Female reproductive outcome

## Abstract

Stress is an important factor that affects physical and mental status of a healthy person disturbing homeostasis of the body. Changes in the lifestyle are one of the major causes that lead to psychological stress. Psychological stress could impact the biology of female reproduction by targeting at the level of ovary, follicle and oocyte. The increased level of stress hormone such as cortisol reduces estradiol production possibly by affecting the granulosa cell functions within the follicle, which results deterioration in oocyte quality. Adaptation of lifestyle behaviours may generate reactive oxygen species (ROS) in the ovary, which further affects female reproduction. Balance between level of ROS and antioxidants within the ovary are important for maintenance of female reproductive health. Physiological level of ROS modulates oocyte functions, while its accumulation leads to oxidative stress (OS). OS triggers apoptosis in majority of germ cells within the ovary and even in ovulated oocytes. Although both mitochondria- as well as death-receptor pathways are involved in oocyte apoptosis, OS-induced mitochondria-mediated pathway plays a major role in the elimination of majority of germ cells from ovary. OS in the follicular fluid deteriorates oocyte quality and reduces reproductive outcome. On the other hand, antioxidants reduce ROS levels and protect against OS-mediated germ cell apoptosis and thereby depletion of germ cells from the ovary. Indeed, OS is one of the major factors that has a direct negative impact on oocyte quality and limits female reproductive outcome in several mammalian species including human.

## Background

Stress is present at every level of society in the form of physical, social and psychological [1]. Lifestyle factors such as psychological stress, cigarette smoking, alcohol consumption, environmental and occupational exposures affect reproductive health of a female [1–3]. The psychological stress may be acute, episodic or chronic depending upon its length and depth of exposure. Acute stress is one of the most common types of psychological stress that occurs mainly due to demands and pressure. Frequent acute stress leads to episodic stress, while endless negative life events results in chronic stress. Reports suggest that chronic psychological stress results in poor in vitro fertilization (IVF) outcome possibly due to its negative impact at the level of ovary and oocytes [4–6].

## Review

### Psychological stress and generation of ROS

Psychological stress due to negative life events lead to direct as well as indirect effects on female reproductive system [5]. Negative life events can directly induce the release of cortisol (a stress hormone), which inhibits estradiol biosynthesis from follicular cells leading to reduced quality and number of retrieved oocytes [5, 7, 8]. It can indirectly impact the reproductive health by changing lifestyle behaviours such as alcohol consumption, cigarette smoking habits etc., that results in the generation of reactive oxygen species (ROS) [5, 9–14]. Alcohol consumption and cigarette smoking have been reported to reduce fertility possibly due to increased level of ROS that causes oxidative stress (OS) [15–18]. Metabolism of alcohol involves formation of nicotinamide adenine dinucleotide (NADH). The NADH induces conversion of xanthine dehydrogenase into its oxidase form that generates superoxide anions and thereby ROS [19, 20]. Smoking causes reduction of molecular oxygen and formation of superoxide radicals. The superoxide radical gets converted either into hydroxyl radical or hydrogen peroxide or both leading to generation of ROS [20]. The increased level of ROS deteriorates oocyte quality by inducing apoptosis [21–25]. There are several other factors that could lead to changes in lifestyle behaviours and generate ROS in a female [9]. Generation of ROS and their clearance through enzymatic and non-enzymatic antioxidants are important in the maintenance of oocyte quality and thereby reproductive health of a female [9, 23, 25–28].

The physiological level of ROS is beneficial during folliculogenesis, oocyte maturation and embryogenesis [10]. Studies from our laboratory suggest that less than 60ng/oocyte is a physiological level of ROS and maintains diplotene arrest in follicular oocytes [22, 29]. Generation of a moderate level of ROS (i.e., 60–80 ng/oocyte) may trigger meiotic resumption from diplotene arrest [22, 29]. The beneficial role of physiological level of ROS is further supported by the observations that the supplementation of antioxidants could inhibit maturation process in rat oocytes cultured in vitro [30]. However, stress can induce production of ROS beyond the physiological range (>80 ng/oocyte) that may result in cell cycle arrest and apoptosis in follicular oocytes [22, 29, 31]. Our studies suggest that increased level of ROS and decreased catalase activity might be involved during final stages of folliculogenesis and oocyte maturation in rat [30, 32]. However, accumulation of ROS beyond physiological level could lead to OS that may deteriorate oocyte quality and thereby affect reproductive outcome [10, 32–37]. The increased cellular metabolism and/or inhibition of enzymatic antioxidants in the ovary may result OS that induces cell cycle arrest and apoptosis both in vivo as well as in vitro [22, 27, 32, 35, 38, 39]. Thus, OS deteriorates oocyte quality, reduces fertilization and pregnancy rates and thereby reproductive outcome in several mammalian species including human [23, 31, 32, 38, 40–42].

## Beneficial role of physiological level of ROS in ovary

The female reproductive function is directly determined by the ovarian life span. Ovary is a metabolically active organ and serves as a germ cell reservoir during reproductive life span of female [43]. It consists approximately 0.3 million primordial follicles that contain diplotene-arrested oocytes [43]. Pituitary gonadotropins surge induces steroidogenesis, follicular growth, development, maturation and ovulation in most of the mammalian species [21, 32, 43]. ROS is generated in the ovary due to increased metabolism during the final stages of folliculogenesis and follicular rupture, which results in the accumulation of ROS level possible due to decreased enzymatic antioxidants activity. This possibility is further supported by the observations that the decrease of catalase activity and increase of hydrogen peroxide as well as total ROS level trigger meiotic resumption from diplotene arrest in rat follicular oocytes [22, 27, 29, 31, 44]. A moderate increase of ROS level favors diplotene-arrested oocytes to resume meiosis inside the follicular microenvironment suggesting the beneficial role of ROS level within the ovary [27, 31, 38, 45, 46]. The high physiological level of ROS has been reported in antral follicles during final stages of folliculogenesis, which could be associated with final maturation of oocytes [21, 30, 47–50].

## Accumulation of ROS leads to OS and deteriorates oocyte quality

Environmental changes, lifestyle changes, pathological conditions or drugs treatment may induce accumulation of ROS leading to OS that may have a negative impact on oocyte physiology by inducing apoptosis [1, 22, 27, 51]. This notion is further strengthened by the observations that the OS caused granulosa cell apoptosis resulting in decreased estradiol 17ß level, ovulation rate and oocyte quality [45]. Studies from our laboratory suggest that OS-mediated granulosa cell apoptosis reduces granulosa cells-oocyte communication that affects supply of nutrients and maturation enabling factors affecting quality of preovulatory oocytes [40]. Further, OS induces shortening of telomere, chromosomal segregation disorders, oocyte fragmentation and fertilization failures thereby age-related decline in fertility [43, 52]. ROS-induced meiotic cell cycle arrest and apoptosis has recently been documented wherein we have proposed that a high ROS level (beyond physiological range) may trigger MPF destabilization and reduction in survival factors leading to mitochondria-mediated oocyte apoptosis [31]. This possibility is further strengthen by our in vitro studies that a transient increase in intracellular ROS leads to meiotic resumption from diplotene arrest, while further increase generates OS that induces cell cycle arrest and apoptosis [22, 29, 53, 54]. Similarly, high level of ROS has been reported to induce cell cycle arrest in human oocytes as well as in mouse embryos [29, 55–57]. Although OS induces cell cycle arrest and apoptosis in immature as well as mature oocytes, immature oocytes are more susceptible and quickly undergo OS-mediated morphological apoptotic changes including shrinkage, membrane blebbing, cytoplasmic granulation and degeneration [22, 41, 58–60]. The repeated ovarian stimulation by exogenous gonadotropin induces OS in the ovary and ovulation of poor quality of oocytes [61]. Both death-receptor as well as mitochondria-mediated pathways are involved in inducing oocyte apoptosis, OS-induced mitochondria-caspase mediated pathway plays a major role in elimination of germ cells from the cohort of ovary and deteriorates oocyte quality even after ovulation [32].

## Prevention and possible treatment against OS

Changes in the lifestyle behaviour due to psychological stress resulting in habit of alcohol consumption, cigarette smoking, etc. induce generation of ROS [5, 9, 10]. Therefore, adaptation of healthy lifestyle as well as avoiding the consumption of alcohol, habit of smoking and use of recreational drugs could reduce level of OS and their negative impact on female reproductive outcome [1, 3, 62]. To minimize OS, antioxidants are useful since they scavenge free radicals and reduce ROS level in the body [63]. Both enzymatic as well as non-enzymatic antioxidants are used to reduce level of ROS and thereby OS [63]. Enzymatic antioxidants such as superoxide dismutase (SOD), catalase, glutathione peroxidase (GPx) and glutathione oxidase and non-enzymatic antioxidants such as vitamin C, taurine, hypotaurine, vitamin E, Zn, selenium (Se), betacarotene, and carotene could be beneficial to overcome this problem [63, 64]. Animal studies suggest that antioxidant supplementation is beneficial to overcome deleterious effects of stress induced OS on mouse oocytes [65]. Melatonin has been considered as one of the potent naturally occurring antioxidants because it prevents OS-mediated deterioration of oocyte quality in rat [23, 27] and improve reproductive outcome in human [66, 67]. Supplementation of daily food practices with different antioxidant providing mediums such as fresh green leafy vegetables, antioxidant rich legumes and plant products may provide support to compensate OS [20].

## Conclusions

**Fig. 1:**
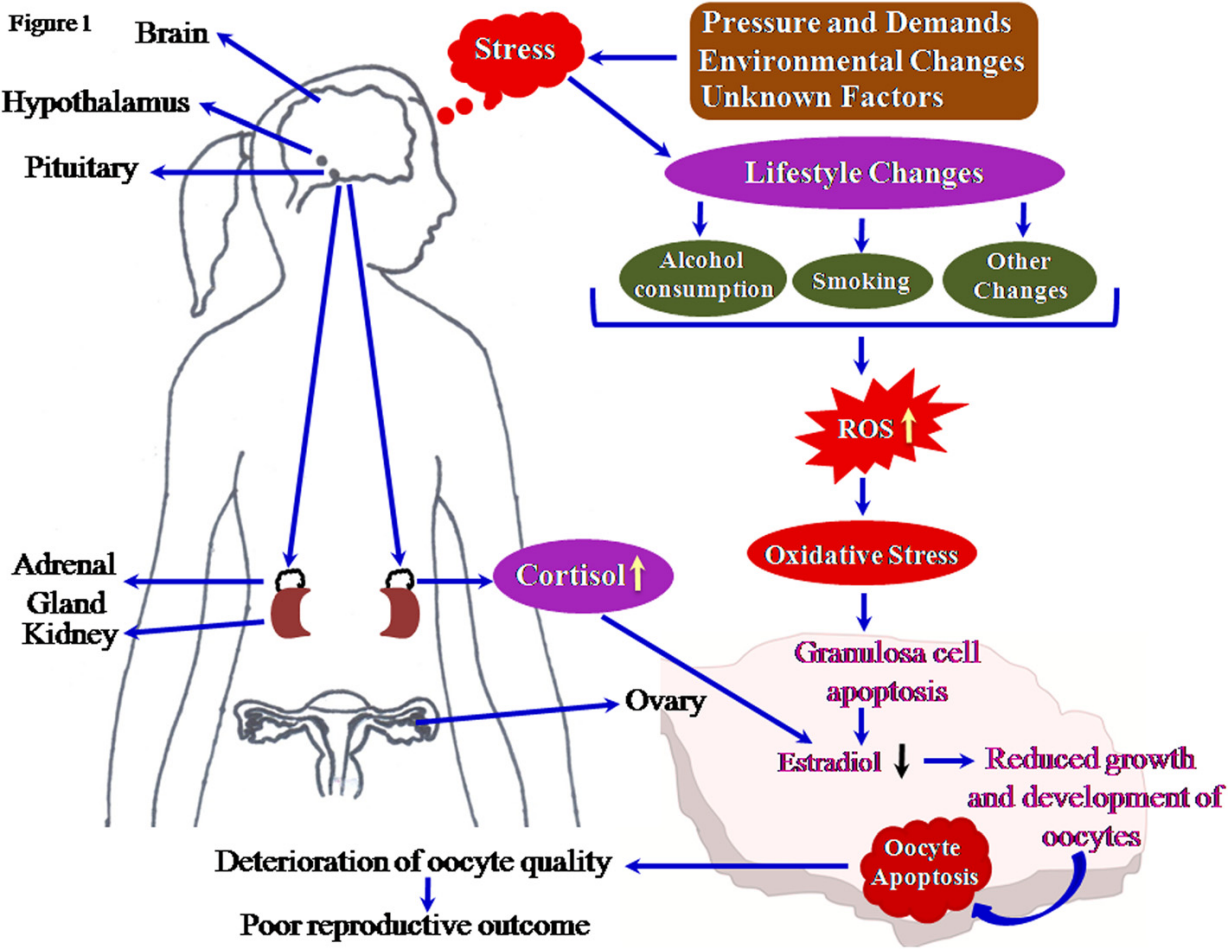
Schematic diagram showing the impact of stress on reproductive outcome. Environmental changes, pressure and demands and several other factors generate psychological stress. The psychological stress induces lifestyle changes and cortisol production from adrenal gland which directly and indirectly affect the ovarian physiology. The lifestyle changes including alcohol consumption and smoking leads to increase level of ROS. The increased level of ROS results an oxidative stress. The increased cortisol as well as oxidative stress levels affects granulosa cell functions possibly by inducing apoptosis. The granulosa cell apoptosis results in reduced estradiol 17ß biosynthesis in the ovary, which reduces growth and development of follicular oocytes and induces oocyte apoptosis. Apoptosis deteriorates oocyte quality leading to poor reproductive outcome in mammals including human

Environmental changes, pressure and demands and several other factors may generate psychological stress. Psychological stress increases production of cortisol that stimulates lifestyle changes. These changes may directly or indirectly affect the physiology of the ovary. Cortisol induces granulosa cell apoptosis and affects estradiol 17ß biosynthesis in the ovary. Reduced level of estradiol in the ovary impairs growth and development of follicles and deteriorates oocyte quality by inducing apoptosis (Fig. 1). High level of ROS in the ovary generates OS, which induces granulosa cell as well as oocyte apoptosis. Indeed, stress could be one of the major causative factors that generates OS, induces apoptosis, deteriorates oocyte quality and reduces reproductive outcome in mammals including human (Table 1). Hence, adaptation of healthy lifestyle, avoiding consumption of alcohol, smoking habit and use of antioxidants as food supplement could be beneficial to overcome stress induced OS-mediated deterioration in oocyte quality and poor reproductive outcome.

**Table 1:** List of studies showing effect of stress on ROS production and oocyte quality

S. No.	Effects of stress on Ros production and oocyte quality	Experimental model	References
1.	Stressful life events cause release stress hormone and impair follicular maturation in ovary	Human	Lancastle and Boivin, 2005 [7], Ebbesen et al., 2009 [5]
2.	Stressful life events leads to maladapted lifestyle behaviours i.e. alcohol and cigarette consumption	Human	Veenstra et al, 2007 [11], Bacharach et al., 2008, Hooper et al., 2008 [13], Ogden et al., 2009[14]
3.	Stress induces poor IVF outcome	Human	Barzilai Pesach et al., 2006 [2], Ebbesen et al., 2009 [5]
4.	Stress generates ROS and reduces oocyte quality	Mice	Kala and Nivsarkar, 2016
5.	alcohol and cigarette consumption reduces fertility in women	Human	Howe et al., 1985 [16], Hakim et al., 1998 [17]
6.	Alcohol consumption increases ROS level	Rat Human	Kato et al., 1990 [19], Videla et al., 2009 [18]
7.	OS triggers oocyte apoptosis	Rat Human	Chaube et al., 2005 [22], 2006 [38], 2008 [53], Tripathi et al., 2011, Tripathi and Chaube, 2012, Tamura et al., 2008
8.	Increase ROS level reduces oocyte quality and reproductive outcome	Rat Human	Chaube et al., 2006, 2008, Tripathi et al., 2011 [27], Tripathi and Chaube, [39]2012, Tamura et al., 2008 [23]

## Abbreviations

GPx: glutathione peroxidase
IVF: In vitro fertilization
NADH: nicotinamide adenine dinucleotide
OS: oxidative Stress
ROS: reactive oxygen species
SOD: superoxide dismutase
